# Gasdermin E Does Not Limit Apoptotic Cell Disassembly by Promoting Early Onset of Secondary Necrosis in Jurkat T Cells and THP-1 Monocytes

**DOI:** 10.3389/fimmu.2018.02842

**Published:** 2018-12-04

**Authors:** Rochelle Tixeira, Bo Shi, Michael A. F. Parkes, Amy L. Hodge, Sarah Caruso, Mark D. Hulett, Amy A. Baxter, Thanh Kha Phan, Ivan K. H. Poon

**Affiliations:** Department of Biochemistry and Genetics, La Trobe Institute for Molecular Science, La Trobe University, Melbourne, VIC, Australia

**Keywords:** DFNA5, secondary necrosis, apoptotic cell disassembly, apoptotic bodies, apoptosis, gasdermin, GSDME

## Abstract

During the progression of necroptosis and pyroptosis, the plasma membrane will become permeabilized through the activation of mixed lineage kinase domain like pseudokinase (MLKL) or gasdermin D (GSDMD), respectively. Recently, the progression of apoptotic cells into secondary necrotic cells following membrane lysis was shown to be regulated by gasdermin E (GSDME, or DFNA5), a process dependent on caspase 3-mediated cleavage of GSDME. Notably, GSDME was also proposed to negatively regulate the disassembly of apoptotic cells into smaller membrane-bound vesicles known as apoptotic bodies (ApoBDs) by promoting earlier onset of membrane permeabilisation. The presence of a process downstream of caspase 3 that would actively drive cell lysis and limit cell disassembly during apoptosis is somewhat surprising as this could favor the release of proinflammatory intracellular contents and hinder efficient clearance of apoptotic materials. In contrast to the latter studies, we present here that GSDME is not involved in regulating secondary necrosis in human T cells and monocytes, and also unlikely in epithelial cells. Furthermore, GSDME is evidently not a negative regulator of apoptotic cell disassembly in our cell models. Thus, the function of GSDME in regulating membrane permeabilization and cell disassembly during apoptosis may be more limited.

## Introduction

Permeabilisation of the plasma membrane during programmed cell death is regulated by a number of distinct molecular factors. For necroptosis, a programmed form of necrosis, plasma membrane permeabilisation is mediated through the phosphorylation and activation of MLKL (mixed lineage kinase domain like pseudokinase) by RIP3/RIP1 necrosome (1). In contrast, plasma membrane permeabilisation during pryoptotic cell death, another form of programmed necrosis, is mediated by caspase 1/4/5 (11)-cleaved GSDMD (gasdermin D) (2). Activation of MLKL and GSDMD can subsequently trigger their targeting toward the plasma membrane through interacting with phosphatidylinositol-4,5-bisphosphate and oligomerisation. This eventually leads to membrane permeabilisation and release of proinflammatory intracellular contents (1, 2). Additionally, it has been shown that, GSDME (gasdermin E, also called DFNA5), a GSDMD-related family member, is activated by caspase 3-mediated cleavage, and its expression level dictates the different forms of cell death (3). Upon caspase 3 activation, GSDME-deficient cells (e.g., GSDME knockout, HeLa cells and Jurkat T cells) first undergo apoptosis followed by secondary necrosis at later stages, whilst cells expressing high GSDME level (e.g., GSDME overexpression, neuroblastoma cells, and skin melanoma cells) proceed rapidly to membrane permeabilisation via similar mechanisms as MLKL and GSDMD (3). The latter phenomenon was also observed in bone marrow-derived macrophages (BMDMs), a cell type that expresses clearly detectable level of GSDME, by Rogers et al. (4). These two studies have challenged the dogma that the progression of apoptotic cells into secondary necrotic cells following membrane permeabilisation is an unregulated process and a consequence of impaired cell clearance. However, it is worth noting that Lee et al. (5) has recently demonstrated that GSDME is not required for secondary necrosis in caspase 1^−/−^ caspase 11^−/−^ BMDMs treated with flagellin, cytochrome c or FasL.

In addition to impaired secondary necrosis, Rogers et al. (4) also reported that the loss of GSDME promoted apoptotic cells to undergo disassembly and generate apoptotic bodies (ApoBDs) (4), suggesting an intriguing inverse relationship between secondary necrosis and ApoBD formation (the latter, a well-known hallmark of apoptosis). We have previously described apoptotic cell disassembly as a highly regulated process controlled by three distinct morphological steps, namely apoptotic membrane blebbing, the formation of thin membrane protrusions known as apoptopodia and beaded apoptopodia, and the detachment of discrete ApoBDs from the apoptotic cell or other ApoBDs (6). In this study we used two established apoptotic cell disassembly models (7, 8) to examine the role of GSDME in regulating the balance between secondary necrosis and ApoBD formation. Interestingly, loss of GSDME expression in both human Jurkat T cells and THP-1 monocytes did not inhibit the progression apoptotic cells into secondary necrotic cells or promote cell disassembly during apoptosis.

## Materials and Methods

### Reagents

Reagents were obtained as follows: trovafloxacin, doxycycline and mitoxantrone (Sigma-Aldrich, MO), anti-Fas (clone CH11, Millipore, MA), annexin A5 (A5)-PE (BD Biosciences, CA) and TO-PRO-3 (Life Technologies, NY).

### Cell Culture

Human Jurkat T cells and THP-1 monocytes were obtained from ATCC and cultured in complete RPMI media. Complete RPMI constituted of RPMI 1640 medium (Life Technologies), 10% (vol/vol) fetal bovine serum (FBS, Bovogen, New Zealand), penicillin (50 U/ml) and streptomycin (50 mg/ml) (Life Technologies), and 0.2% (vol/vol) MycoZap (Lonza, Switzerland). Human A431 squamous epithelial cells (Lonza) were cultured in MEM (Lonza) supplemented with 10% FBS, penicillin (50 U/ml) and streptomycin (50 mg/ml), and non-essential amino acid and L-glutamine (Thermofisher Scientific, MA).

#### CRIPSR/Cas9 Gene Editing

A doxycycline-inducible sgRNA vector CRISPR/Cas9 system was used to generate gene disruptions as previously described (9). Jurkat T and THP-1 cells stably expressing Cas9 endonuclease and mCherry were generated by lentiviral transduction using pFUCas9mCherry plasmid. GSDME targeting sgRNA were generated by annealing the following oligonucleotides 5′ TCCCGTCGGACTTTGTGAAATACG-3′ and 5′AAACCGTATTTCACAAAGTCCGAC-3′, and ligating into pFgh1tUTG plasmid. Jurkat T and THP-1 cells expressing Cas9 and mCherry were single cell sorted using FACS Aria II (BD Biosciences). The stably-Cas9-expressing cells were then infected with lentiviral supernatant containing GSDME targeting sgRNA pFgh1tUTG constructs. Jurkat T cells were treated with doxycycline (1 μg/ml) treatment for 72 h and mCherry (indicative of Cas9) and GFP (indicative of sgRNA) positive cells were single cell sorted using FACS Aria II. For THP-1 cells, mCherry and GFP positive cells were bulk sorted using FACS Aria II and treated with doxycycline (1 μg/ml) for 7 days.

### Immunoblotting

Samples were lysed at 4°C in lysis buffer [20 mM HEPES pH 7.4, 1%, IGEPAL® CA-630, 10% glycerol, 1% Triton X-100, 150 mM NaCl, 50 mM NaF, protease inhibitor cocktail tablet (Roche, CH)], analyzed by SDS-PAGE and immunoblotted using the following antibody dilutions: anti-GSDME (1:1,000, clone EPR19859, Abcam), anti-ERK2 (1:1,000, clone D-2, Santa Cruz), anti-β actin? (1:4,000, clone AV-15, Sigma-Aldrich), in 3% BSA in PBST (0.1% Tween). Blots were incubated in secondary HRP-conjugated donkey anti-rabbit (1:5,000, Millennium Science, AU) or sheep anti-mouse (1:5,000, Millennium Science) antibodies in 5% milk in PBST (0.1% Tween). HRP signal was developed using ECL (GE Lifesciences) and captured using the Syngene G:Box gel documentation and analysis system (Syngene, MD).

### Induction of Apoptosis

Cells in 1% BSA or complete RPMI were induced to undergo apoptosis with UV irradiation (150 mJ/cm^2^) using Stratagene UV Stratalinker 1800 (Agilent Technologies, CA) and incubated at 37°C in humidified atmosphere with 5% CO_2_ for 4 h or as indicated. In certain experiments as indicated, apoptosis was induced with anti-Fas treatment (0.5 μg/ml, Merck, Germany). For A431 cells, apoptosis was also induced using mitoxantrone (4 ng/ml, Sigma-Aldrich, MO) and incubated at 37°C in humidified atmosphere with 5% CO_2_ for 4–6 h.

### Microscopy

Cells were seeded in a 4 or 8 chambered Nunc™ Lab-Tek™ II chambered coverglass (Nunc, Denmark) prior to induction of apoptosis and drug treatment. For Jurkat T and THP-1 cells, chambers were pre-treated with poly-L-lysine (Sigma Aldrich). A431 cells were seeded 16 h prior to induction of apoptosis. Time-lapse differential interference contrast imaging was performed at 37°C with 5% CO_2_ using Spinning Disc Confocal microscope (Zeiss, Germany) with × 63 oil immersion objective. Image processing and analysis was performed using Zeiss imaging software (Zeiss).

### Flow Cytometry Analysis of Cell Viability, Cell Lysis, and ApoBD Formation

Samples were stained with A5-PE (1:200 dilution) and TO-PRO-3 (0.5 μM) in 1 × A5 binding buffer (BD Biosciences) at room temperature (RT) in dark for 10 min. Samples were analyzed using FACSCanto II Flow cytometer (BD Biosciences). Data analysis was performed using FlowJo software (version 9.8.5, FlowJo, OR) as previously described (10). Briefly, necrotic cell (TO-PRO-3^high^) was separated from other events (TO-PRO-3^low/intermidate^). The latter population was further gated into two groups: (i) SSC^high^ A5^low/intermediate^, used to identify viable cells (FSC^high^ TO-PRO-3^low^), and (ii) the remaining events, for further determination of apoptotic cells (FSC^high^ A5^high^) and ApoBDs (FSC^low^ A5^intermedidate/high^). The level of ApoBD formation was reflected by ApoBD formation index, the ratio between ApoBDs and apoptotic cells in the sample.

### Lactate Dehydrogenase (LDH) Release Assay

LDH release assay was performed as previously described (11) using a LDH Cytotoxic Assay Kit II (Abcam). Briefly, cell lysis was determined by detecting release of cytosolic LDH in culture supernatants. Cells were induced to undergo apoptosis by UV irradiation or anti-Fas treatment in a 96 well plate in complete RPMI at 37°C, 5% CO_2_ in humidified atmosphere for either 4 or 16 h. Cell suspensions were centrifuged at 300 g for 10 min to remove cells and the supernatant was centrifuged at 3,000 *g* for 20 min to remove cell debris. Resultant supernatant was added to LDH reaction mix for 30 min at RT. Absorbance was measured at 450 nm using SpecraMax M5e Plate reader (Molecular Devices, CA) and data was analyzed using SoftMaxPro 5.2 software (Molecular Devices).

### Statistics

Data is represented as + s.e.m. Statistical significance was determined using One-way analysis of variance (ANOVA) followed by Turkey *post-hoc* test or, where appropriate, unpaired students' two-tailed *T*-test. *P* < 0.05 were considered significant. ^*^P < 0.05, ^**^P < 0.01, ^***^P < 0.001.

## Results

**Figure 1:**
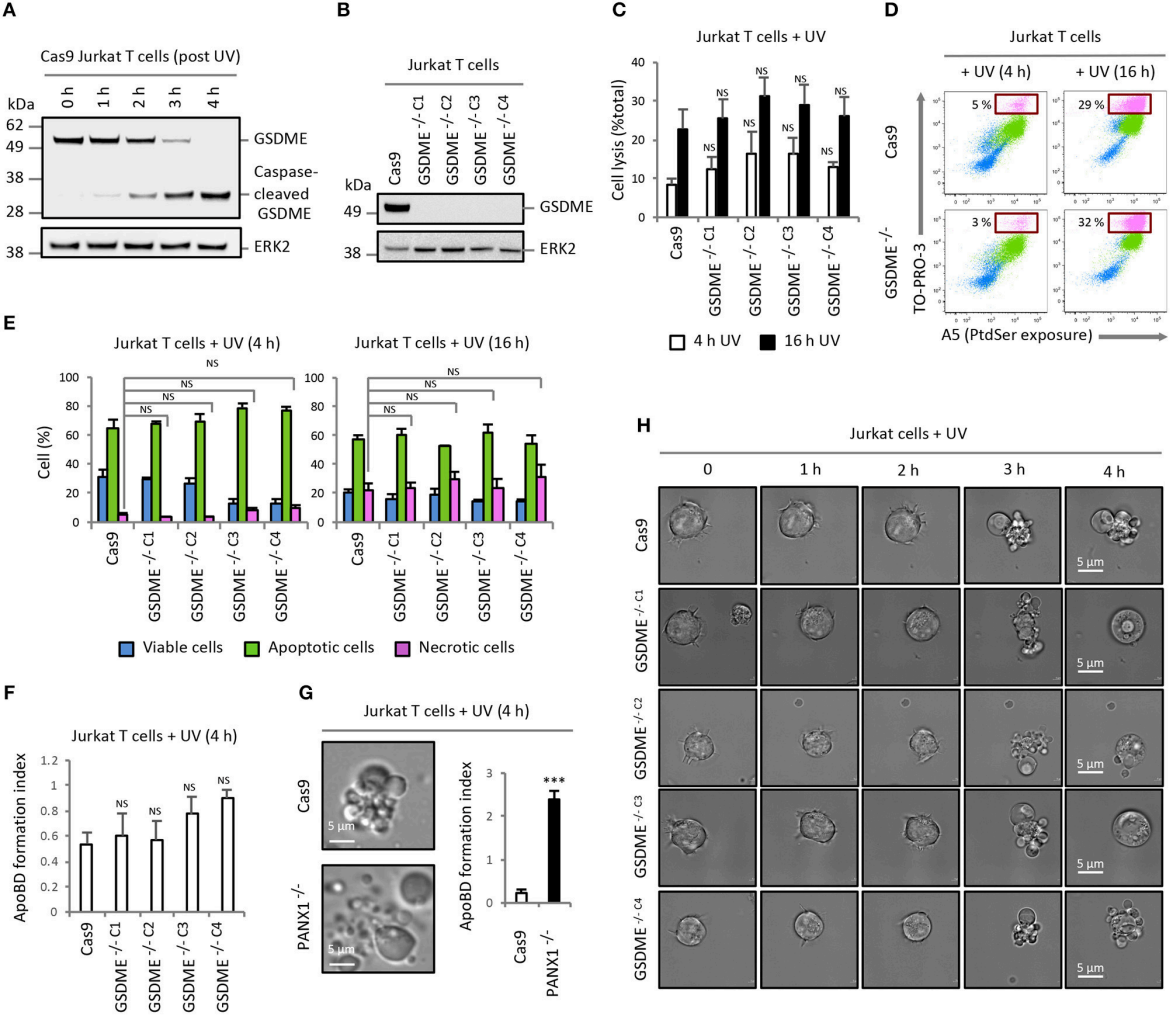
Loss of GSDME does not affect the level of secondary necrosis and ApoBD formation in Jurkat T cells. **(A)** Expression of GSDME and proteolytic processing of GSDME during UV-induced apoptosis (150 mJ/cm^2^) in Cas9 Jurkat T cells. **(B)** Loss of GSDME protein expression with CRISPR/Cas9-mediated *GSDME* gene disruption in Jurkat T cell clonal populations. GSDME expression in **(A,B)** were detected using immunoblotting analysis. **(C)** Levels of cell lysis in Cas9 control and GSDME^−/−^ Jurkat T cells treated with UV irradiation was quantified based on the release of LDH into the culture supernatant (*n* = 3). **(D)** Representative flow cytometry plots of viable, apoptotic and necrotic cells generated by Cas9 control and GSDME^−/−^ Jurkat T cells treated with UV irradiation to induce apoptosis. **(E)** Levels of viable, apoptotic and necrotic cells in Cas9 control and GSDME^−/−^ Jurkat T cells treated with UV irradiation to induce apoptosis was determined by flow cytometry (*n* = 3). **(F)** Formation of ApoBDs from apoptotic Cas9 control and GSDME^−/−^ Jurkat T cells (*n* = 3). ApoBD formation index determined by the number of ApoBDs divided by the number of A5^+^ apoptotic cells. **(G)** Disassembly of apoptotic Cas9 and PANX1^−/−^ Jurkat T cells was monitored by live DIC microscopy and flow cytometry (*n* = 3). **(H)** Live DIC microscopy images monitoring morphologies of UV-irradiated Cas9 control and GSDME^−/−^ Jurkat T cells over 4 h. Error bars represent s.e.m. Data are representative of at least two independent experiments. *P-*values were determined by directly comparing a GSDME^−/−^ clone to Cas9 control at that particular timepoint, using One-way ANOVA *post-hoc* using Turkey's test in **(C,E,F)** or unpaired Student's two-tailed *t-*test in **(G)**. ***P < 0.001, NS = *P* > 0.05.

The expression of GSDME was detected in human Jurkat T cells, and induction of apoptosis by UV irradiation promoted the generation of a GSDME fragment at ~35 kDa that corresponded to the caspase-cleaved GSDME noted in previous studies (3, 4) (Figure 1A). To investigate the role of GSDME in membrane permeabilisation and cell disassembly during apoptosis, we generated GSDME^−/−^ Jurkat T cells by CRISPR/Cas9-based gene editing approach (Figure 1B and additional GSDME^−/−^ Jurkat T cell lines shown in Figure S1A). We then determined whether loss of GSDME will lead to a reduction in Jurkat T cells progressing to secondary necrosis upon apoptotic stimulation by monitoring the release of the cytosolic protein lactate dehydrogenase (LDH) into the culture supernatant [also used in (3, 4)]. Surprisingly, all GSDME^−/−^ Jurkat T cell lines exhibited similar levels of necrotic lysis as Cas9 control cells at 4 and 16 h post-apoptosis induction by UV (Figure 1C and Figure S1B) or anti-Fas treatment (Figure S2). To quantify the progression of apoptosis, we performed flow cytometry analysis using A5 (detect exposure of phosphatidylserine) and TO-PRO-3 (membrane-impermeable nucleic acid stain, only entering cells through caspase 3/7-activated plasma membrane channel pannexin 1 (PANX1) during early stages of apoptosis or upon membrane permeabilisation). Comparable levels of necrosis (TO-PRO-3^high^ A5^high^ cells) were consistently detected in Cas9 control and GSDME^−/−^ Jurkat T cells (Figures 1D,E and Figure S1C).

Furthermore, using our recently established multi-parametric gating strategy (10) on the flow cytometry dataset, we were able to quantify and compare the level of ApoBD formation by apoptotic Cas9 and GSDME^−/−^ cells. Unexpectedly, GSDME^−/−^ Jurkat T cell lines were found to generate similar levels of ApoBDs as Cas9 control cells (Figure 1F and Figure S1D), suggesting that GSDME is not a negative regulator of the apoptotic cell disassembly process in this cell model. In contrast, loss of PANX1, a previously described negative regulator of ApoBD formation (8), in Jurkat T cells markedly promoted the generation of ApoBDs upon UV (Figure 1G) or anti-Fas treatment (Figure S3). Next, we used time-lapse differential interference contrast (DIC) microscopy to visualize our observations and confirmed that Cas9 control and GSDME^−/−^ Jurkat T cells were able to display typical apoptotic morphologies such as dynamic membrane blebbing over 4 h post apoptosis induction without apparent effect on secondary necrosis (Figure 1H and Videos 1, 2).

**Figure 2:**
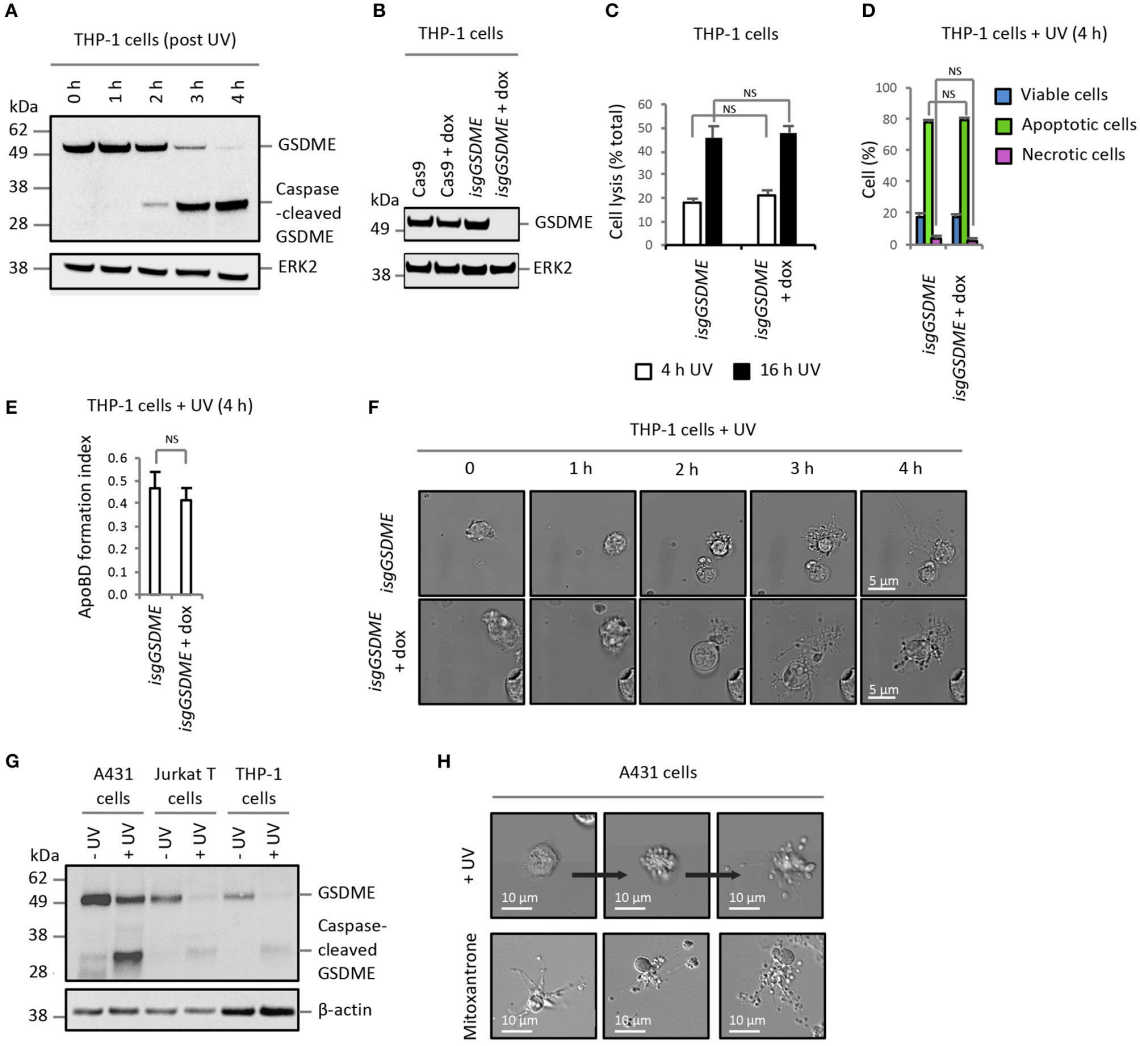
Expression level of GSDME does not alter secondary necrosis and ApoBD formation in other cell models**. (A)** Expression of GSDME and proteolytic processing of GSDME during UV-induced apoptosis (150 mJ/cm^2^) in THP-1 monocytes. **(B)** Loss of GSDME protein expression with CRISPR/Cas9-mediated *GSDME* gene disruption upon doxycycline (dox) treatment of *isgGSDME* THP-1 cells. **(C)** Levels of cell lysis, reflected by LDH release, in untreated and dox-treated *isgGSDME* THP-1 cells (*n* = 3). Flow cytometry analysis showing **(D)** the levels of viable, apoptotic and necrotic cells and **(E)** ApoBD formation index of UV-irradiated untreated and dox-treated *isgGSDME* THP-1 cells (*n* = 3). **(F)** Live DIC imaging of untreated and dox-treated *isgGSDME* THP-1 cells over 4 h post-UV irradiation. **(G)** Expression of GSDME and proteolytic processing of GSDME during UV-induced apoptosis (150 mJ/cm^2^) in A431 epithelial cells, in comparison to Jurkat T and THP-1 cells. GSDME expression in **(A,B,G)** were detected using immunoblotting analysis. **(H)** Live DIC imaging of A431 cells treated with UV or mitoxantrone (4 ng/mL, 5 h) to induce apoptosis. Error bars represent s.e.m. Data are representative of at least three independent experiments. NS = *P* > 0.05, unpaired Student's two-tailed *t*-test.

Similar results were also observed for THP-1 monocytic cells, in which GSDME is evidently expressed and cleaved during apoptosis (Figure 2A). Compared to control THP-1 cells (untreated *isgGSDME*), THP-1 cells deficient in GSDME (*isgGSDME* + dox; Figure 2B) did not show a reduction in the level of membrane permeabilised cells (Figures 2C,D) or enhanced cell disassembly during apoptosis (Figures 2E,F). Furthermore, A431 epithelial cells (expressing a relatively higher level of GSDME than Jurkat T cells and THP-1 monocytes, Figure 2G) induced to undergo apoptosis by UV-irradiation or mitoxantrone treatment can readily undergo apoptotic cell disassembly (12) (Figure 2H). Collectively, these data suggest that GSDME is dispensable for secondary necrosis and is not a negative regulator of apoptotic cell disassembly in Jurkat T and THP-1 monocytic cells. Furthermore, caspase-mediated activation of GSDME during apoptosis does not limit apoptotic cell disassembly in A431 epithelial cells.

## Discussion

It is important to understand the mechanistic basis of programmed necrosis as the exposure of intracellular contents is linked to autoimmune response, inflammation and tissue injury (13–15). In this study, we show that GSDME is not required for cells to progress into secondary necrosis and does not negatively regulate apoptotic cell disassembly. One might attribute the discrepancy between previous studies (3, 4) and this study to the cell type-dependent role of GSDME, which may correlate with its expression level. However, while we cannot eliminate this possibility, several lines of evidence do question the previously proposed role of GSDME in apoptosis. Even though GSDME was expressed at a lower level in Jurkat T and THP-1 monocytic cells as compared to A431 epithelial cells, expression and cleavage of GSDME during apoptosis were clearly evident in these cell types. Whether a yet to be identified mechanism(s) is present in these cell types/lines that could limit the proposed function of GSDME remains to be determined. In addition, it is worth noting that although GSDME^−/−^ bone marrow-derived macrophages [a cell type that expresses clearly detectable level of GSDME (4, 5)]was initially found to exhibit an impairment in undergoing secondary necrosis following treatment with etoposide or vesicular stomatitis virus (4), recent studies reported GSDME is not required for secondary necrosis in caspase 1^−/−^ caspase 11^−/−^ bone marrow-derived macrophages treated with flagellin, cytochrome c or FasL (5). Thus, GSDME-mediated secondary necrosis may not be a predominate consequence of apoptosis and may only occur under specific conditions for a certain cell type. In support of the former concept, there is ample evidence in the literature demonstrating the ability of apoptotic T cells (8), thymocytes (8, 16), B cells (17), monocytes (7), fibroblasts (8), smooth muscle cells (18), epithelial cells (12), and endothelial cells (19) to exhibit morphological hallmarks of apoptosis including membrane blebbing and ApoBD formation without early onset of secondary necrosis to disrupt the progression of cell disassembly.

As described earlier, apoptotic cell disassembly is generally regulated by three sequential morphological steps including membrane blebbing, thin membrane protrusion formation and cell fragmentation into ApoBDs (6). It is therefore logical to argue that if membrane permeabilisation does occur prior to the final cell fragmentation step, earlier onset of secondary necrosis driven by any mechanism could negatively regulate ApoBD formation. However, from the point of view of apoptotic cell clearance, it is surprising that a process downstream of caspase 3 (e.g. GSDME cleavage) would actively drive early onset of secondary necrosis, an undesirable event during apoptosis. In fact, apoptotic cells prevent membrane permeabilisation mediated through, for example, pyroptosis (cleavage of GSDMD by caspase 3 to generate non-membrane lytic form of GSDMD) (20). Simultaneously, apoptotic cells also release “find-me” signals and expose “eat-me” signals to promote cell clearance by phagocytes to limit secondary necrosis (21, 22). It is important to acknowledge that we are still at the early stages in understanding the function of GSDME, however, in the context described in this study, GSDME does not regulate secondary necrosis or function as a negative regulator of apoptotic cell disassembly.

## Author Contributions

RT, BS, MP, TP and IP designed, performed, and analyzed most of the experiments with help and input from AH, SC, MH, and AB. IP, TP, and RT wrote the manuscript with input from co-authors.

### Conflict of Interest Statement

The authors declare that the research was conducted in the absence of any commercial or financial relationships that could be construed as a potential conflict of interest.
