# Supplementary figures and images for: Gasdermin E Does Not Limit Apoptotic Cell Disassembly by Promoting Early Onset of Secondary Necrosis in Jurkat T Cells and THP-1 Monocytes
Source: Front Immunol. 2018 Dec 4;9:2842. doi: 10.3389/fimmu.2018.02842 (PMC6288192; doi:10.3389/fimmu.2018.02842)

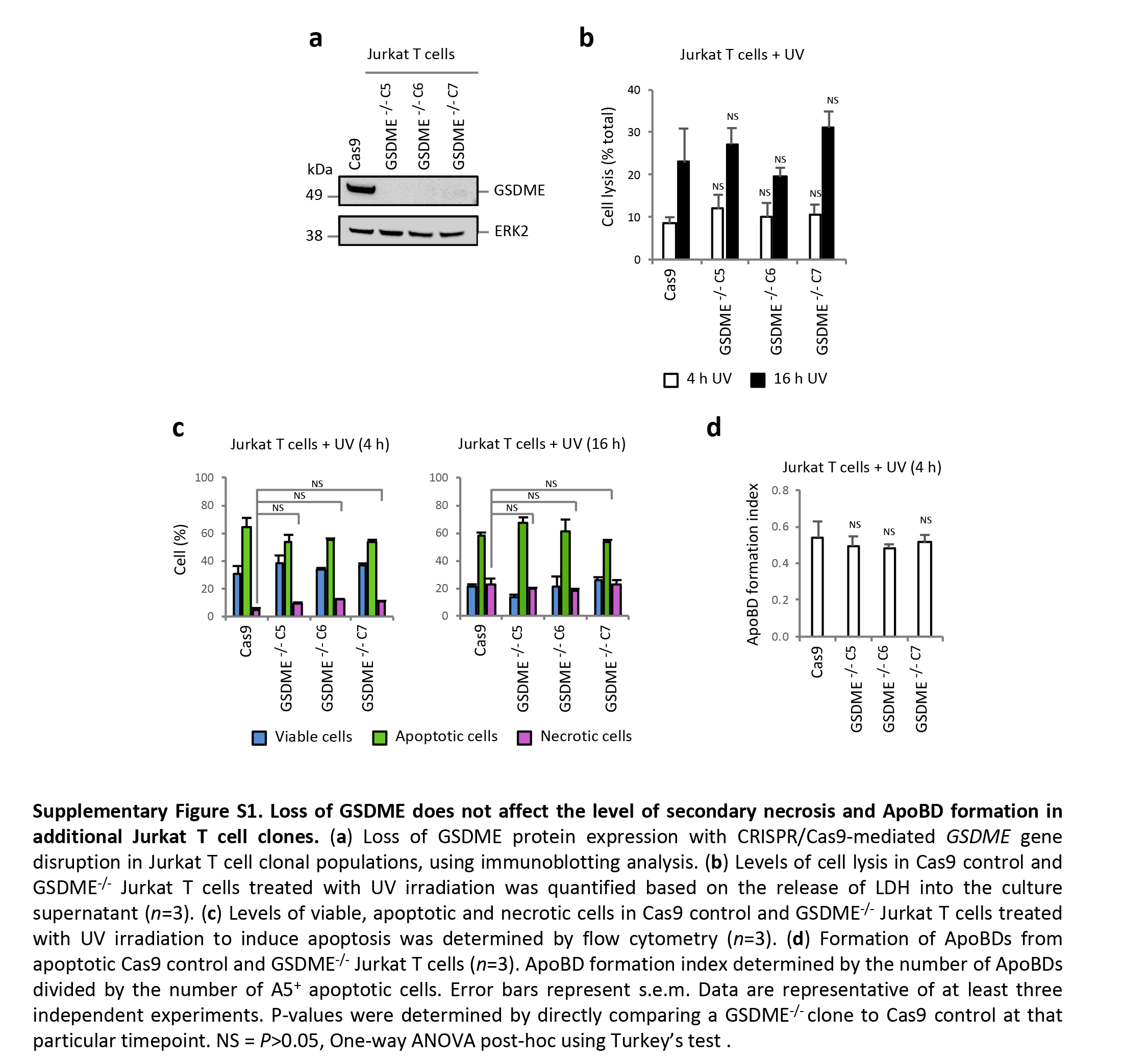

Supplement: Supplementary file 1 [file Image_1.JPEG]

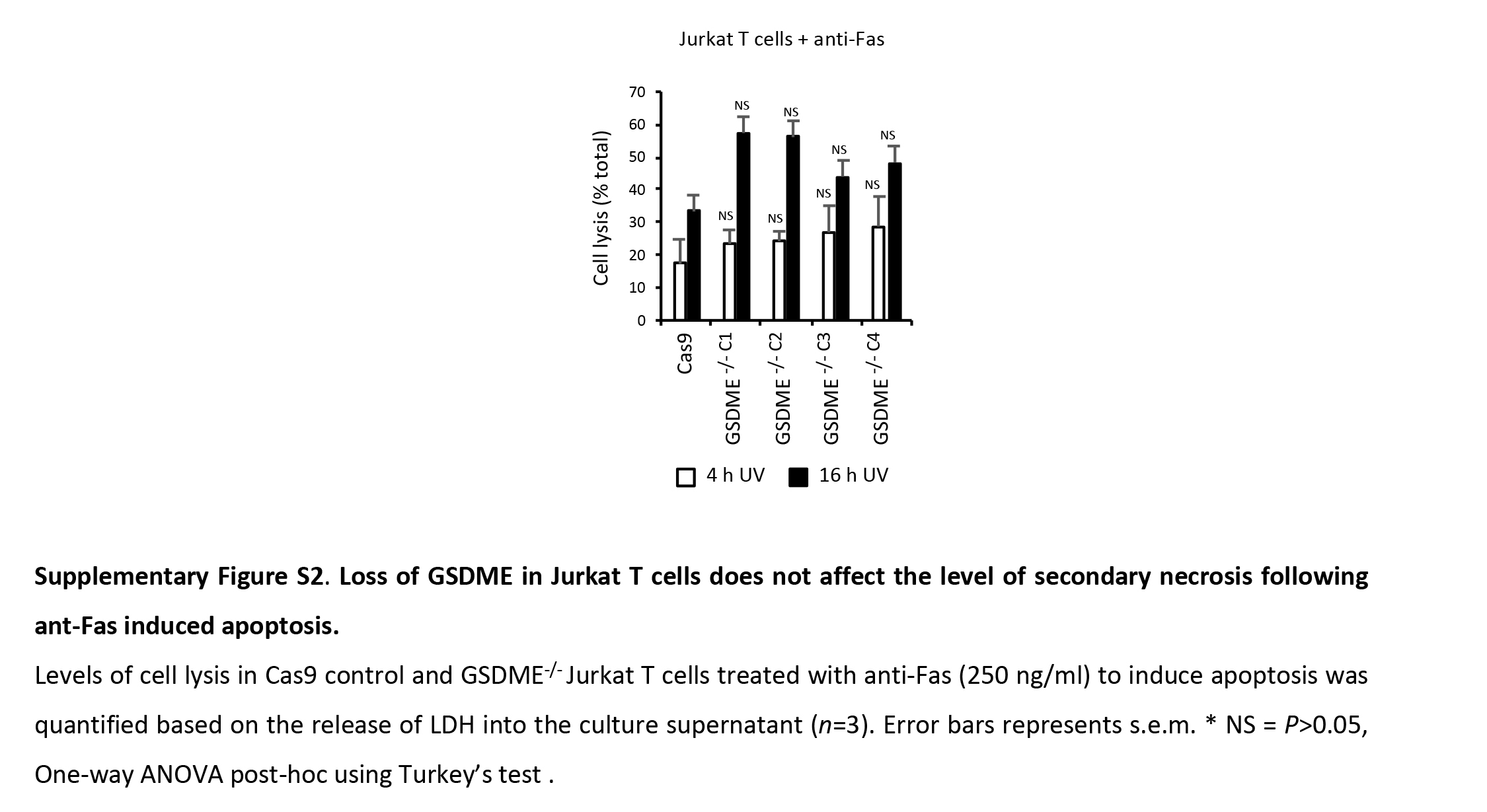

Supplement: Supplementary file 2 [file Image_2.JPEG]

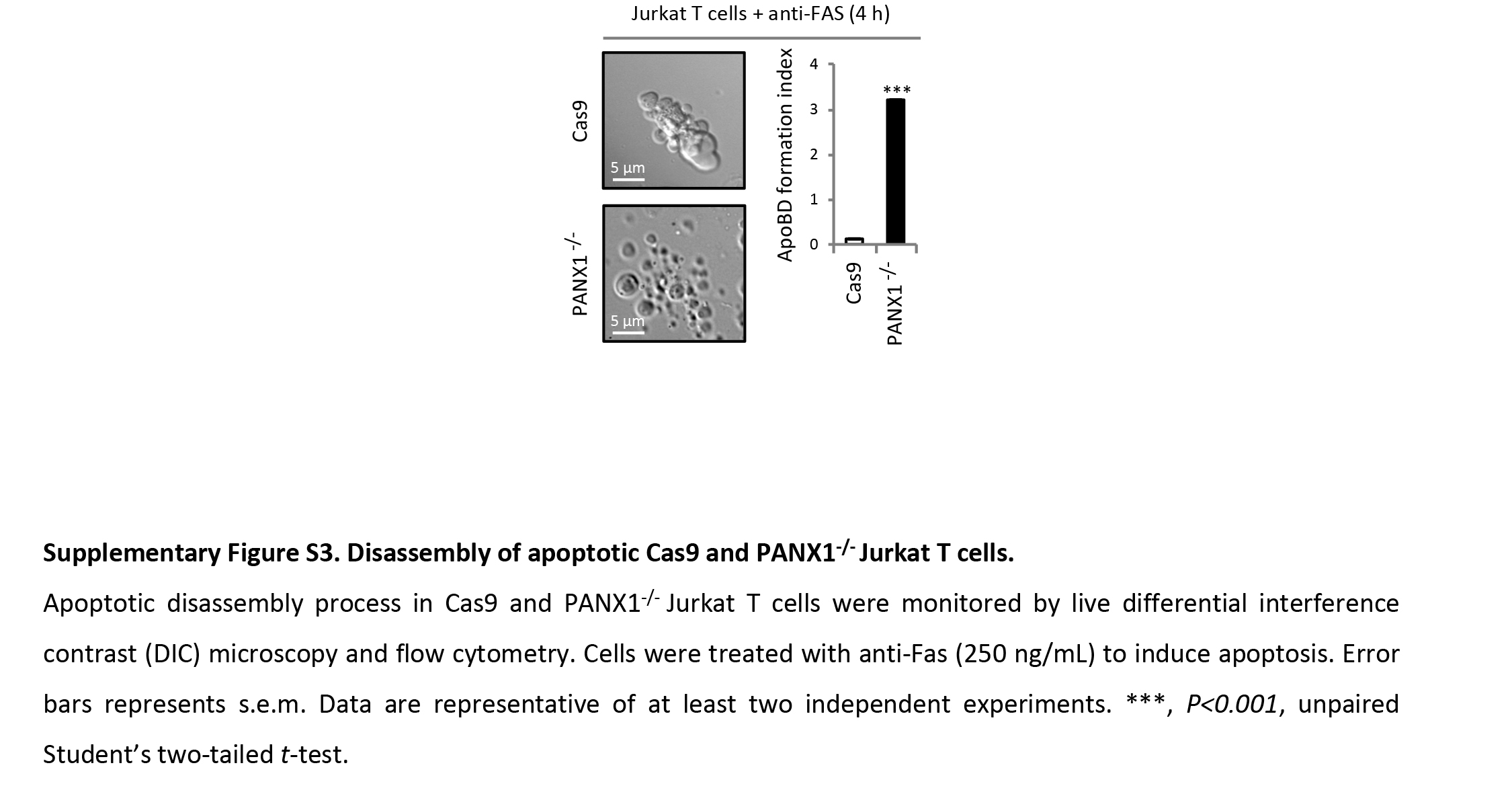

Supplement: Supplementary file 3 [file Image_3.JPEG]
